# Epstein–Barr Virus: More than Just an Infection

**DOI:** 10.3390/v18091048

**Published:** 2026-09-21

**Authors:** Evelyn Van de Perre, Brieuc Van Nieuwenhuyse, Jean Cyr Yombi

**Affiliations:** 1 Faculty of Medicine, UCLouvain, 59 Promenade de l’Alma, 1200 Brussels, Belgium; evelyn.vandeperre@student.uclouvain.be; 2 Department of Internal Medicine and Infectious Diseases, Cliniques Universitaires Saint-Luc, UCLouvain, 10 Avenue Hippocrate, 1200 Brussels, Belgium

**Keywords:** Epstein–Barr virus, viral latency, multiple sclerosis, autoimmune diseases, hemophagocytic lymphohistiocytosis (HLH), lymphoproliferative disorders, post-transplant lymphoproliferative disorder (PTLD), chronic active Epstein–Barr virus disease (CAEBV)

## Abstract

While typically associated with benign infectious mononucleosis, Epstein–Barr virus (EBV) is now recognized as a pivotal driver of complex immunopathological disorders. By shuttling between lytic replication and strategic latency programs, EBV reprograms B-cell biology to evade immune surveillance and disrupt homeostasis. Central to this pathogenesis is the viral protein EBNA1, which triggers autoimmunity through molecular mimicry with host antigens, including GlialCAM in multiple sclerosis (MS), keratin in rheumatoid arthritis, and nuclear proteins in systemic lupus erythematosus. These autoimmune responses are further exacerbated by bystander activation and epitope spreading, fueling chronic inflammation in Crohn’s disease, type 1 diabetes, and IgA nephropathy. EBV can also promote the development of lymphoproliferative disorders, whether in immunocompetent hosts or in the context of immunosuppression; however, the emergence of EBV-positive forms within diseases that are usually EBV-negative is closely linked to an underlying state of immunosuppression. Distinct from these chronic states, EBV-associated hemophagocytic lymphohistiocytosis (HLH) represents a critical hyperinflammatory emergency in which defective cytotoxicity leads to an uncontrolled T-cell- or NK-cell-driven cytokine storm; together with chronic active EBV disease (CAEBV), HLH disproportionately affects children, and both receive particular attention in this review. More recently, emerging but not yet consolidated evidence has also suggested a possible association between EBV reactivation and long COVID. Elucidating these diverse molecular interactions is essential for developing the next generation of targeted therapies and vaccine strategies aimed at mitigating the systemic impact of this virus.

## 1. Introduction

Historically, viruses have primarily been viewed through the lens of their acute infectious potential. However, accumulating experimental and epidemiological evidence has progressively revealed their involvement in complex, and at times chronic, pathophysiological processes [1].

Epstein–Barr virus (EBV) is a particularly illustrative example of this paradigm shift. Classically identified as the causative agent of infectious mononucleosis [1,2], it is now recognized for its association with several conditions, including certain neoplasms [1], autoimmune diseases [3], multiple sclerosis [4], specific forms of hemophagocytic lymphohistiocytosis [5,6], certain lymphoproliferative disorders [7], and, more recently, an emerging and still-debated association with long COVID [8].

This evolving understanding now calls for moving beyond the concept of the virus as a mere infectious agent, toward viewing it as a potential modulator situated at the core of immunological and cellular mechanisms implicated in a range of diseases.

This article provides a review of the literature on EBV. In light of current evidence, we examine the molecular and immunological mechanisms by which EBV may contribute to the pathophysiology of several non-infectious diseases, and open discussion on its potential as a therapeutic or preventive target. Particular emphasis is placed on hemophagocytic lymphohistiocytosis (HLH) and chronic active EBV disease (CAEBV), two conditions with a marked pediatric specificity that is often underrepresented in adult-focused reviews of EBV pathology.

Although first identified as an oncogenic virus, EBV has gained increasing relevance across other areas of medicine, driven by a growing understanding of its interaction with the immune system, particularly in autoimmune disease. Although individually rare, EBV-associated conditions collectively represent a substantial global public health burden, making this ubiquitous virus a driving force behind novel prophylactic and therapeutic strategies.

## 2. Literature Search Strategy and Scope

This is a non-systematic narrative review. Relevant literature was identified through PubMed/MEDLINE searches (final search date: August 2026) combining the terms “Epstein-Barr virus” with “neoplasm”, “multiple sclerosis”, “autoimmune disease”, “hemophagocytic lymphohistiocytosis”, “lymphoproliferative disorder”, “post-transplant lymphoproliferative disorder”, “chronic active Epstein-Barr virus disease”, and “long COVID”, restricted to English- and French-language publications. Priority was given to systematic reviews, meta-analyses, large prospective cohorts, and landmark mechanistic studies published within the last decade, supplemented by earlier foundational references where historically relevant. Reference lists of retrieved articles were hand-searched for additional sources. Given the narrative (non-systematic) design, no formal protocol was registered and no formal risk-of-bias assessment was performed, consistent with the Scale for the Assessment of Narrative Review Articles (SANRA) guidelines for the conduct and reporting of narrative reviews; the level of evidence therefore varies across sections and is summarized qualitatively in Table 1 and discussed further in Section 13.

## 3. Epidemiology

EBV infection is highly prevalent worldwide. An estimated 95% of the population carries anti-EBV antibodies [3]. In the current absence of an approved vaccine, this seropositivity necessarily reflects a prior encounter between the virus and the immune system, i.e., a past infection. Seroprevalence increases with age [3]: each additional year of life has been shown to be associated with a 12% increase in the risk of EBV infection [3].

The age at which primary infection occurs, however, varies according to a country’s level of development [3]. In low-resource countries, primary infection often occurs during early childhood and is reflected in a sharp early rise in seroprevalence; low income and crowded domestic living conditions are contributing factors [3]. In Europe and other high-resource countries, the rise in seroprevalence is more gradual: a seroprevalence of 90% is only reached by age 22 [3].

## 4. Pathophysiology

### 4.1. Primary Infection: Transmission and Route of Entry

Oral transmission via saliva is the principal route of EBV transmission; primary infection targets the oropharynx, with viral tropism for both epithelial cells and B lymphocytes [1,7]. Which cell type is infected first remains debated, with competing models proposing either a primary epithelial infection or direct B-cell infection [1].

### 4.2. Dual Viral Cycle: The Productive Lytic Phase and the Latent Phase

Following infection, EBV establishes lifelong latency in memory B lymphocytes [1,4,7], with possible reactivation. Its life cycle alternates between a lytic cycle, which ensures replication, and a latent cycle, which ensures persistence, governed by distinct gene expression programs [1,4,7].

There are four latency types, correlated with the differentiation stage of the infected B lymphocyte, each defined by a distinct pattern of non-productive gene expression that ensures survival, proliferation, and persistence without lytic replication (summarized in Table 2) [1,4,7]. The latency repertoire comprises nine latency proteins—six nuclear antigens (EBNA-1, -2, -3A, -3B, -3C, -LP) and three membrane proteins (LMP-1, -2A, -2B)—together with non-coding RNAs (BART and BHRF1 microRNAs, and EBERs).

### 4.3. Chronological Dynamics

During primary infection, infected epithelial cells drive primary lytic replication [1], while infected B lymphocytes activate a latency III program (proliferation), expressing the full complement of latency genes [1,4].

Most of these cells are eliminated by NK cells and the primary T-cell response. A fraction progress to the germinal center under a latency II (default) program, characterized by restricted gene expression that favors cell survival [7] and immune evasion [1,4,7].

Over the long term, latency persists under the latency 0 program, characterized by near-total transcriptional quiescence [1,4,7], following migration of the B lymphocytes to a circulating memory phenotype with no detectable immune response [1,7]. Upon cell division, these cells switch to latency I, expressing EBNA1 to transmit the viral genome to daughter cells [1,4].

EBV reactivation remains possible and requires recruitment of infected memory B lymphocytes into the germinal center [1], without necessarily producing virions [1,4]. These cells then either return to dormancy or differentiate into virus-shedding plasma cells [1].

The four latency programs predominate in EBV-associated oncogenesis, but viral replication itself constitutes an independent pathogenic factor [1].

### 4.4. EBV Pathogenesis: Key Molecular Concepts

EBV pathogenesis is best understood through its latency products: nuclear antigens (EBNA1, 2, 3A–C, LP), membrane proteins (LMP1, 2), and non-coding RNAs (BHRF1/BART miRNAs, EBERs), which hijack B-lymphocyte homeostasis and underlie the pathophysiological mechanisms of the disease (detailed in Table 3) [1,4,7].

## 5. EBNA1: The Achilles’ Heel of EBV

Unlike proviruses, the EBV genome persists during latency as an episome—a circular, nucleosomal DNA molecule lacking a centromere [13] and is therefore unable to rely on standard cellular segregation machinery [13,14]. EBNA1 compensates for this limitation: its DNA-binding domain tethers the episome to host chromosomes during mitosis, while its homodimer binds palindromic sites within oriP—the FR region (segregation) and the DS region (replication) [13,14].

This dual role makes EBNA1 the cornerstone of viral persistence, a prerequisite for oncogenic processes [14,15]. It is expressed in virtually all virus-associated tumors, except in latency 0 (where no cell division occurs) [14]; in certain B-cell malignancies restricted to latency I, it is in fact the only detectable viral protein [14,15].

This near-universal tumor expression has given rise to a therapeutic hypothesis: blocking EBNA1 would progressively dilute viral episomes across successive mitoses [15,16], potentially offering a broadly applicable weapon against EBV-positive cancers [15,16].

Until 2018, no viable candidate molecule had emerged, owing to insufficient sensitivity and specificity in vitro [15], although this work validated the efficacy of EBNA1 inhibition and the feasibility of targeting it [15].

In 2019, a team at the Wistar Institute designed, using a structure-based approach, the first inhibitors of the EBNA1 DNA-binding interface, reducing viral load and tumor growth in preclinical models [16]. The mechanism, suspected to be allosteric, remains to be elucidated; in vivo efficacy, evaluated only in murine xenografts, requires further toxicological validation and combination with other therapies [16].

This transition to the clinic culminated in a first-in-human phase I trial of the oral inhibitor VK-2019 in 23 patients with advanced nasopharyngeal carcinoma, confirming a favorable safety profile [17]. A reduction in EBV genome copies and EBER-1 levels demonstrated targeted biological activity, but clinical efficacy as monotherapy remained modest, and the original dose-escalation trial was subsequently discontinued for lack of efficacy. Development has since been redirected toward new phase 2 trials in nasopharyngeal carcinoma and, more recently, a phase 1b trial in relapsed or refractory EBV-positive diffuse large B-cell lymphoma, reflecting a broader strategy of combination regimens and alternative EBV-driven malignancies rather than abandonment of EBNA1 as a target.

## 6. Infectious Mononucleosis

Infectious mononucleosis (IM) represents the most characteristic acute clinical presentation of primary EBV infection. This symptomatic presentation is not, however, universal: some primary infections remain asymptomatic, while others may present atypically. Age at the time of infection is the principal determinant of the risk of developing clinical IM [2,3].

Primary infections occurring during adolescence or young adulthood are more likely to present with the classic clinical picture than those occurring at other ages. Primary EBV infection in adolescents and young adults results in classic mononucleosis in 70–75% of cases [2,3]. Although the clinical presentation of primary infection in younger children has historically been less extensively characterized than in adolescents, a retrospective cohort of children aged 0–15 years tested for suspected primary EBV infection found that about two-thirds presented with a recognizable mononucleosis-like syndrome, while the remaining third had an atypical or oligosymptomatic presentation, which was more common at younger ages [18]. At the population level, the proportion of infections accompanied by clinically apparent mononucleosis rises sharply around the age of puberty, with the cumulative risk of infectious mononucleosis before age 30 estimated at 13% in males and 22% in females [19].

Clinically, IM is classically characterized by a symptomatic triad of fever persisting for two to four weeks, pharyngitis, and cervical lymphadenopathy [2]. These manifestations typically appear after an incubation period of four to seven weeks [2]. The great majority of cases resolve spontaneously, but acute complications, although rare, have been reported, including hepatitis, splenic rupture, and acute respiratory distress secondary to tonsillar hypertrophy [2].

This symptomatic triad guides the diagnosis, but confirmation relies on laboratory testing. The heterophile antibody test (Monospot), used as first-line testing, has a sensitivity of 63–84% and a specificity of 84–100% [2]; false negatives are common during the first week of symptoms, and up to 10% of affected patients never develop a positive test. When diagnostic doubt persists, EBV-specific serology is useful: anti-VCA IgM and IgG are informative in the acute phase, while anti-EBNA antibodies help distinguish recent from past infection; these tests, although more sensitive and specific than the heterophile test, are costlier and slower to obtain [2].

To date, no specific antiviral treatment has demonstrated clinical benefit. Management therefore remains essentially symptomatic: relative rest, adequate hydration, and simple antipyretics and analgesics form the mainstays of treatment.

## 7. EBV and Multiple Sclerosis

Multiple sclerosis (MS) is a chronic, autoimmune, demyelinating inflammatory disease of the central nervous system. Although the hypothesis of a causal microbial agent, first proposed by Charcot in 1868, has never been confirmed for any single direct pathogen, EBV now appears to be a necessary but not sufficient factor for its development [20]. This relationship rests on a body of converging evidence: near-universal EBV seroprevalence among MS patients (up to 99%) [4]; a longitudinal study of more than 10 million US military personnel showing a 32-fold increase in MS risk following EBV infection—with no comparable effect for other viruses such as CMV—associated with a rise in serum neurofilament light-chain levels only after seroconversion [21]; and a significantly amplified anti-EBV humoral and T-cell response in MS patients, detailed in Table 4 [4]. The best-supported pathophysiological mechanism is molecular mimicry: Lanz et al. showed that anti-EBNA1 antibodies display strong cross-reactivity with GlialCAM, a central nervous system protein, providing a mechanistic explanation for the breakdown of immune tolerance characteristic of MS [22]. The absence of any difference in viral load between individuals who later develop MS and controls, both in peripheral blood and cerebrospinal fluid, suggests that the role of EBV does not stem from direct viral toxicity but rather from long-term immune dysregulation in predisposed individuals—a dysregulation that is amplified when primary infection presents late in life as infectious mononucleosis. Because EBV represents an obligatory step in this process, its prevention theoretically offers an opportunity to avert new cases, which underlies the current interest in an EBV vaccine, although such programs remain at an early stage [4].

This near-deterministic model has recently been nuanced. A population-based study using province-wide laboratory records from Ontario, Canada, identified 74 cases in which MS onset preceded a first documented primary EBV infection (confirmed by heterophile antibody or viral capsid antigen IgM testing), a substantially larger number than expected from the original military cohort alone [23]. Possible explanations include diagnostic misclassification, false-negative or delayed EBV testing, or genuine etiological heterogeneity within the clinical entity currently labeled as MS; these findings argue against using EBV serology as a rule-out criterion in diagnostic algorithms at this stage. In parallel, high and persistently elevated anti-EBNA1 peptide antibody titers have been shown to help distinguish MS from other neuroinflammatory diseases such as MOGAD (myelin oligodendrocyte glycoprotein antibody-associated disease) and neuromyelitis optica spectrum disorder, suggesting a possible future role for serial EBV serology as a diagnostic adjunct rather than a purely etiological marker [24].

### Vaccine Candidates in Development

Several EBV vaccine candidates are currently in clinical development. The most advanced, mRNA-1189 (Moderna), uses mRNA technology encoding five envelope glycoproteins (gp350, gB, gH, gL, gp42) involved in viral entry; a phase 1/2 trial (ClinicalTrials.gov identifier: NCT05164094; now extended to adolescents as young as 10 years of age) aims to prevent primary infection and mononucleosis; as of this writing, the trial remains ongoing and no efficacy results have yet been reported. Other approaches, focused on gp350 alone or on EBNA1, remain preclinical or in early-phase testing; no candidate has yet demonstrated protective efficacy, and the optimal target antigen remains uncertain.

## 8. EBV and Autoimmunity Beyond Multiple Sclerosis

Beyond MS, EBV has been associated with several other autoimmune diseases (rheumatoid arthritis, systemic lupus erythematosus, inflammatory bowel disease, IgA nephropathy, type 1 diabetes, juvenile idiopathic arthritis, celiac disease), based on increased seroprevalence among affected patients, a correlation between anti-EBV antibody titers and clinical severity, and the detection of viral DNA and latency proteins in affected tissues [3]. This relationship fits within a multifactorial model in which EBV acts both as a trigger in genetically predisposed individuals—a predisposition dominated by class II HLA alleles, notably HLA-DRB1*15:01, HLA-DQB1*06:02, and HLA-E*01:01—and as a modulator of that predisposition, through four mechanisms summarized in Table 5 [3]: molecular mimicry, i.e., structural homology between viral proteins (EBNA1, EBNA2, gp110) and self-antigens (joint proteins, nuclear antigens Sm B/SmD3/Ro60/C1q) generating cross-reactivity, as illustrated in rheumatoid arthritis and systemic lupus erythematosus [25]; bystander activation, i.e., antigen-independent lymphocyte activation by pro-inflammatory cytokines released during infection, contributing to inflammatory bowel disease via TLR9 and IL-17A [26]; epitope spreading, whereby cell lysis releases normally sequestered intracellular antigens and broadens the immune response to new self-epitopes, as suggested in IgA nephropathy through increased production of galactose-deficient IgA1 [27]; and epigenetic transactivation, whereby the latency protein EBNA2 acts as a transcription factor at host susceptibility loci, sustaining chronic immune activation [3]. Vaccine-based prevention of these autoimmune diseases by targeting EBV remains hypothetical, given the lack of evidence for an obligatory causal role comparable to that demonstrated in MS, and carries a theoretical risk—through the very molecular mimicry mechanism it would seek to prevent—of precipitating an autoimmune process in predisposed individuals if the chosen vaccine antigen, such as EBNA1, is itself implicated in this mechanism. This mechanistic picture has recently moved from correlation toward direct demonstration: single-cell profiling has shown that EBV can directly reprogram autoreactive, antinuclear-antigen-specific B cells into activated antigen-presenting cells in patients with systemic lupus erythematosus, providing a plausible cellular mechanism by which the virus sustains chronic autoimmune activation rather than merely correlating with it [28,29].

## 9. EBV and Hemophagocytic Lymphohistiocytosis (HLH)

Hemophagocytic lymphohistiocytosis (HLH) is a potentially fatal acute hyperinflammatory syndrome (“cytokine storm”) resulting from uncontrolled activation of cytotoxic T lymphocytes and NK cells together with pathological hemophagocytosis by activated tissue macrophages. EBV is the infectious agent most frequently associated with HLH, although HLH remains a rare complication of infection, and other viruses (HIV, CMV) or no identifiable trigger may also be responsible [5]. A distinction is made between primary (genetic) HLH, which typically affects children with a family history or recurrent episodes, and secondary HLH, without a purely genetic etiology, which arises through a “two-hit” mechanism in older children, adolescents, or adults, most often following infection or in the context of malignancy or autoimmune disease; X-linked lymphoproliferative syndrome (XLP, or Purtilo syndrome) constitutes a distinct form, with the XLP1 subtype (SH2D1A mutation) almost exclusively associated with EBV, and the XLP2 subtype (XIAP/BIRC4 deficiency) predisposing to recurrence and severe inflammatory bowel disease [5]. Differentiation from infectious mononucleosis relies on the intensity and duration of symptoms: unlike the self-limited fever of IM, which typically resolves within two to four weeks, HLH is characterized by unremitting fever persisting beyond 7 days despite adequate antimicrobial therapy, together with marked hepatosplenomegaly and neurological involvement, with coagulopathy and cytopenias further pointing toward HLH; suspicion of the diagnosis warrants measurement of fibrinogen, triglycerides, ferritin, soluble IL-2 receptor (sCD25), and, in children, CXCL9 and IL-18. The H-score, which outperforms the HLH-2004 criteria (sensitivity 90%, specificity 79%), helps formalize the diagnosis, without overlooking other possible etiologies (neoplastic, infectious, autoimmune, rheumatologic) [5,6]; revised 2024 diagnostic criteria have since removed the requirement for NK-cell function testing, reflecting its limited availability and reproducibility in routine practice. Because the cellular reservoir of EBV differs between patients, therapeutic strategy is tailored to viral tropism: in B-cell-driven EBV-HLH, rituximab depletes the infected B-cell reservoir and rapidly reduces viral load, whereas a persistently elevated EBV PCR despite adequate B-cell depletion should raise suspicion of underlying T- or NK-cell infection, for which rituximab alone is not an adequate standalone therapy. In T-/NK-cell-driven EBV-HLH, standard HLH-94/HLH-2004 immunochemotherapy (etoposide and dexamethasone) or JAK1/2 inhibition with ruxolitinib constitutes the backbone of treatment, with antivirals playing only a secondary role in either scenario. For refractory disease irrespective of cell tropism, targeted anti-cytokine agents are increasingly used as second-line or salvage therapy: ruxolitinib dampens the hyperinflammatory cascade downstream of multiple cytokines, while emapalumab, an anti-interferon-γ monoclonal antibody approved for primary HLH, has shown encouraging results in case series of EBV-HLH, particularly as a bridge to allogeneic transplantation, although comparative trial data specific to the EBV-driven form remain limited [30]. The overarching goal remains to reduce viral stimulation while controlling hyperinflammation with appropriate immunomodulatory therapies [5].

Because primary HLH is overwhelmingly a disease of infancy and childhood, most large EBV-HLH cohorts are pediatric by design. A multicenter retrospective study of 264 children with EBV-HLH across six centers in China developed a baseline-characteristics prognostic score that stratified patients into three risk groups with significantly different survival, underscoring both the heterogeneity of outcomes and the value of early risk stratification in this age group [31]. The geographic and ethnic distribution of EBV-HLH is not confined to East Asia, however: although historically over-represented in East Asian and Hispanic cohorts, a substantial proportion of cases also occur in European and North American populations of diverse ancestry, suggesting that susceptibility is not restricted to a specific geographic or ethnic background and may instead reflect a combination of environmental EBV exposure patterns and genetic modifiers of NK-cell cytotoxicity [32]. Compared with EBV-HLH in adults, pediatric presentations more frequently reveal an underlying primary immune deficiency, most notably X-linked lymphoproliferative syndrome, so a first episode in a child should systematically prompt screening for such a genetic predisposition, since its presence alters both prognosis and the threshold for proceeding to allogeneic transplantation [33]. First-line treatment in children still largely follows the HLH-94/HLH-2004 protocols (etoposide and dexamethasone, with cyclosporine A added from week 9), which achieve a 5-year survival of approximately 60% [34]; more recently, response-adapted regimens incorporating the JAK1/2 inhibitor ruxolitinib have achieved faster remission and substantially reduced etoposide and glucocorticoid exposure specifically in pediatric EBV-HLH, without compromising short-term survival, positioning this approach as a promising less-toxic alternative pending confirmation in prospective trials [35].

## 10. EBV and Lymphoproliferative Disorders

Primary infection results in EBV persistence within memory B lymphocytes, where latency programs 0 and I (restricted gene expression) allow escape from immune surveillance in an immunocompetent host. When this surveillance is impaired (immunodeficiency), EBV can reactivate the immunogenic and oncogenic latency II and III programs (Table 2 and Table 3), whose activation can lead to EBV-positive lymphoproliferative disorders; according to Chapman et al. [7], an additional genetic “second hit” is generally still required for malignant transformation.

It is important to note that lymphoproliferative disorders can also arise in the context of a latency I expression program, in which case multiple associated genetic events are required [7].

The 5th edition of the WHO classification of EBV-associated lymphoproliferative disorders(Table 6) distinguishes three categories: disorders that are consistently EBV-positive and arise in immunocompetent hosts (lymphomatoid granulomatosis, EBV-positive diffuse large B-cell lymphoma, possibly related to immunosenescence); disorders that are also consistently EBV-positive but specific to a context of immunosuppression; and lymphomas that are more often EBV-negative but may present an EBV-positive subgroup, which the WHO now proposes to identify given its close association with immunosuppression [5]. Age also stratifies this landscape within the T- and NK-cell lineage in particular: the WHO and International Consensus Classification systems recognize several entities that are essentially confined to children and young adults, such as systemic EBV-positive T-cell lymphoma of childhood and hydroa vacciniforme lymphoproliferative disorder, as distinct from their adult-predominant counterparts, extranodal NK/T-cell lymphoma and aggressive NK-cell leukemia [36].

### 10.1. Post-Transplant Lymphoproliferative Disorders (PTLD)

Post-transplant lymphoproliferative disorders (PTLD) are iatrogenic clonal lymphoid proliferations occurring after solid organ transplantation (SOT) or allogeneic hematopoietic stem cell transplantation (allo-HSCT), regardless of the time elapsed since transplantation; their main risk factors—type of transplant, transplanted organ, HLA compatibility, intensity of T-cell immunosuppression, and donor/recipient EBV serostatus—are detailed in Table 7 [9]. In the large majority of cases, PTLD results from malignant transformation of B lymphocytes latently infected with EBV, whose proliferation escapes the immune surveillance normally provided by cytotoxic T lymphocytes when this is compromised by post-transplant immunosuppression; however, approximately 20–40% of cases, mainly late-onset monomorphic forms, are EBV-negative [9]. Clinically, presentation is heterogeneous and often misleading, and may mimic infectious mononucleosis (fever, asthenia, lymphadenopathy), which requires keeping the immunosuppressive context in mind when considering the diagnosis [9]. Diagnosis relies on histopathological examination, which classifies the disease according to the 2022 WHO/ICC criteria into four categories with direct therapeutic implications (non-destructive, monomorphic, polymorphic, and classic Hodgkin lymphoma forms), detailed in Table 8, and should be supplemented by metabolic imaging (18F-FDG PET-CT), a complete biological and hemostasis work-up, quantification of EBV and CMV viral load by PCR, and bone marrow biopsy [9]. In addition to the management of established PTLD, preemptive monitoring of EBV viral load with early administration of rituximab in patients with rising EBV DNAemia is widely used to prevent progression to overt PTLD; pooled data suggest response rates on the order of 90% when rituximab is used preemptively versus approximately 65% once PTLD is established, and a large solid-organ-transplant cohort has confirmed an association between rituximab exposure and a reduction in PTLD incidence over time, particularly in patients with high-risk serostatus or lung/multivisceral grafts, although prospective randomized confirmation is still lacking [37]. Once PTLD is diagnosed, treatment, guided by histological subtype, follows a risk-stratified sequential algorithm—first-line reduction of immunosuppression, followed by weekly rituximab, then R-CHOP-type immunochemotherapy in cases of insufficient response—as antivirals and immunoglobulins are ineffective against latent EBV; targeted approaches such as tabelecleucel, an allogeneic off-the-shelf EBV-specific T-lymphocyte therapy, have been approved for refractory forms in the European Union, the United Kingdom, and Switzerland since 2022 on the basis of the phase 3 ALLELE trial (objective response rate approximately 51%) [9,38]; as of mid-2026, however, the therapy has twice been denied approval by the US FDA, which cited concerns about the design and interpretability of the pivotal trial rather than safety or manufacturing issues, illustrating the persistent gap between regulatory jurisdictions for rare EBV-driven diseases.

### 10.2. EBV-Associated Epithelial Malignancies

Beyond lymphoproliferative disease, EBV is also a major driver of epithelial malignancy. Nasopharyngeal carcinoma (NPC), consistently EBV-positive and endemic to Southern China and Southeast Asia, accounts for an estimated 119,750 new cases annually worldwide [11]. EBV-associated gastric cancer, a distinct molecular subtype recognized by the WHO classification of gastric tumors, is present in approximately 7–10% of gastric adenocarcinomas and is estimated to account for roughly 81,000 cases per year globally, making it one of the most common EBV-associated malignancies worldwide [12]. Both entities differ mechanistically from the lymphoproliferative disorders discussed above, reflecting a restricted, non-lytic latency II program in malignant epithelial cells rather than B-lymphocyte transformation, and are not further detailed in this review, which focuses on EBV-associated lymphoproliferative and hyperinflammatory disease.

## 11. Chronic Active Epstein–Barr Virus Disease (CAEBV)

Chronic active EBV disease (CAEBV) is a rare, potentially fatal lymphoproliferative syndrome affecting T and/or NK lymphocytes, reclassified by the WHO in 2022 as a proliferative entity in its own right rather than a simple persistent infection; it mainly affects children and young adults, with a rising incidence in adults, and predominates in East Asia and among Indigenous populations of Latin America, with a predisposing genetic background (HLA-DQB1 allele) suspected [10]. Pathophysiologically, the classic pediatric model describes an initial infection of bone marrow hematopoietic progenitors with establishment of type II latency (EBNA1, LMP1, LMP2) in T or NK lymphocytes, LMP1 activating the NF-κB and JAK/STAT pathways and promoting cell proliferation and immortalization; in adults, an alternative model suggests direct infection of mature T/NK lymphocytes carrying acquired genetic alterations, with viral strains harboring deletions that favor “leaky” expression of the lytic gene BZLF1, giving rise to genomic instability conducive to clonal expansion and tumor progression [10]. The clinical picture combines persistent or recurrent mononucleosis-like symptoms (fever, lymphadenopathy, hepatosplenomegaly) lasting more than 3 months, which may be complicated by severe visceral involvement (uveitis, central nervous system involvement, interstitial lung disease, myocarditis, coronary artery aneurysms), or may progress to HLH or T/NK-cell leukemic or lymphomatous transformation if left untreated. Diagnosis relies on four criteria—persistent symptoms beyond 3 months, high EBV viral load (>10,000 IU/mL), demonstration of EBV infection in T or NK lymphocytes (EBER-positive cells), and exclusion of a pre-existing immunodeficiency or lymphoma [10]. Conventional antivirals and chemotherapy or corticosteroid therapy alone provide only transient control; hematopoietic stem cell transplantation with reduced-intensity conditioning remains the only validated curative option, with a 3-year survival of 70–90% in Asia compared with approximately 55% at 2 years in Western countries, while emerging approaches (EBV-specific cytotoxic T lymphocytes, JAK1/2 inhibitors, checkpoint inhibitors) remain investigational. A phase 2 trial of the JAK1/2 inhibitor ruxolitinib in systemic CAEBV reported complete responses in approximately one-fifth of patients, with most responders able to proceed to allogeneic transplantation with better-controlled disease activity, positioning ruxolitinib as a potential bridge-to-transplant strategy rather than a standalone curative option [39].

CAEBV was first described, and remains best characterized, in children and young adults in East Asia, where the disease typically follows a more indolent, protracted course than the rare adult-onset forms. In this population, the central management challenge is timing allogeneic transplantation early enough to prevent irreversible organ damage while avoiding unnecessary transplant-related risk in patients who might otherwise remain stable for longer. A long-term single-center study following 52 pediatric and young-adult patients over two decades in Japan found that the size of the dominant EBV-infected T- or NK-cell subpopulation carried prognostic value for guiding this timing decision, supporting a more individualized, biology-based approach to transplant indication rather than reliance on clinical severity alone [10,40].

## 12. EBV and Long COVID

Beyond its oncological and autoimmune associations, EBV has also been implicated in the pathophysiology of long COVID. Several studies published since 2021 report EBV reactivation (positivity for anti-EA-D IgG or anti-VCA IgM antibodies) in a substantial proportion of patients with persistent symptoms following SARS-CoV-2 infection, associated with more pronounced fatigue and neurocognitive symptoms [8]. These findings suggest that some manifestations of long COVID may result from viral reactivation triggered by COVID-19-related inflammation rather than from a direct effect of SARS-CoV-2; this emerging field requires confirmation through larger prospective studies.

## 13. Limitations of This Review

This review has several limitations. It is a non-systematic narrative review, without a standardized protocol (e.g., PRISMA) or formal assessment of the methodological quality of the cited studies, exposing it to a risk of selection bias. The level of evidence varies across conditions: the link with MS is supported by prospective cohorts encompassing several million individuals, whereas the association with type 1 diabetes, celiac disease, or juvenile idiopathic arthritis relies on a smaller body of evidence subject to residual confounding. This field is also evolving rapidly: several of the data cited here (ongoing vaccine trials, incidence of EBV-associated cancers) may quickly become outdated.

## 14. Conclusions

In conclusion, Epstein–Barr virus is far more than a simple infection. Beyond the homeostatic disruptions of primary infection—infectious mononucleosis or, more rarely, HLH—it is responsible for a persistent post-viral syndrome grounded in its capacity to establish chronic latency. Through the expression of its latency products, EBV durably disrupts the host’s genetic, cellular, immunological, and molecular homeostasis. These changes, silent in most individuals, create in some a favorable ground for the emergence of chronic conditions such as multiple sclerosis, certain autoimmune diseases, or lymphoproliferative disorders.

Current knowledge has established EBV as a major target for the development of therapeutic and preventive strategies, paving the way for the first steps toward their clinical implementation. However, further scientific advances remain necessary to deepen our understanding of the underlying mechanisms and pathophysiological patterns involved, a prerequisite for the effective translation of these approaches into clinical practice.

On the one hand, such progress is needed to refine the development of intervention strategies while ensuring the efficacy and safety of the approaches considered. On the other hand, continued research is essential to inform public health decisions, particularly regarding assessment of the cost–benefit ratio of proposed interventions.

## 15. Practical Recommendations

Several practical principles emerge from the evidence reviewed above. EBV viral load should be measured whenever a lymphoproliferative disorder is suspected or an immunocompromised patient presents with unexplained prolonged fever. In solid organ or hematopoietic stem cell transplant recipients, EBV viral load should be monitored regularly, with immunosuppression reduced as soon as it becomes detectable, in order to help prevent PTLD. Atypical, severe, or prolonged infectious mononucleosis should prompt consideration of chronic active EBV disease and referral of the patient to a specialized center. Clinicians caring for patients from nasopharyngeal carcinoma- or gastric cancer-endemic regions should likewise remain alert to EBV-associated epithelial neoplasms, to support timely recognition and referral. Finally, patients should be informed of the potential role of EBV in certain autoimmune diseases, particularly multiple sclerosis, given the current absence of a validated antiviral treatment or vaccine.

## Figures and Tables

**Table 1:** Overview of the major EBV-associated conditions discussed in this review, their proposed pathogenic mechanism(s), and the corresponding level of supporting evidence [1,3,4,5,6,7,8].

Disease/Condition	Proposed EBV-Related Mechanism(s)	Level of Supporting Evidence
Multiple sclerosis (MS) [4]	Molecular mimicry (anti-EBNA1 antibody cross-reactivity with GlialCAM); near-universal seroprevalence; EBV infection precedes disease onset	Strong—large prospective cohorts (>10 million subjects) and consistent mechanistic data
Other autoimmune diseases (RA, SLE, IBD, IgA nephropathy, T1D, JIA, celiac disease) [3]	Molecular mimicry, bystander activation, epitope spreading, and epigenetic transactivation (EBNA2)	Moderate to limited—smaller cohorts, correlative data, residual confounding
Hemophagocytic lymphohistiocytosis (HLH) [5,6]	Uncontrolled cytotoxic T-cell/NK-cell activation, arising either from EBV infection of B lymphocytes or from direct EBV infection of T or NK lymphocytes Genetic background	Moderate—well-characterized clinical syndrome; EBV is the most common but not the only trigger
EBV-positive lymphoproliferative disorders [7]	Reactivation of immunogenic/oncogenic latency II-III programs under impaired immunosurveillance, generally requiring a genetic “second hit”	Strong—well-defined histopathological entities per WHO classification
Post-transplant lymphoproliferative disorder (PTLD) [9]	Loss of cytotoxic T-cell immunosurveillance under iatrogenic immunosuppression, allowing outgrowth of latently infected B cells	Strong—large transplant-cohort data with well-defined risk factors
Chronic active EBV disease (CAEBV) [10]	Aberrant latency II infection of T or NK lymphocytes, with genomic instability in adult-onset forms	Moderate—mainly case series and regional cohorts; rare disease
Long COVID [8]	Reactivation of latent EBV secondary to SARS-CoV-2-related inflammation	Emerging/limited—early observational studies; causality not established
Epithelial malignancies (NPC, gastric cancer) [11,12]	Reactivation of latent EBV in a restricted, non-lytic latency II program within infected epithelial cells, promoting oncogenic transformation	Strong—well-defined histopathological entities; large population-based cancer-registry data (IARC GLOBOCAN 2022)

CAEBV: chronic active Epstein–Barr virus disease; HLH: hemophagocytic lymphohistiocytosis; IBD: inflammatory bowel disease; JIA: juvenile idiopathic arthritis; MS: multiple sclerosis; PTLD: post-transplant lymphoproliferative disorder; RA: rheumatoid arthritis; SLE: systemic lupus erythematosus; T1D: type 1 diabetes.

**Table 2:** This table summarizes the different EBV latency programs, highlighting the plasticity of viral gene expression according to the B-lymphocyte differentiation cycle. It correlates each latency type with its predominant biological function, as well as the associated protein and non-coding RNA expression profiles [1].

Latency Program	Program Name	Differentiation Stage (Infected B Cell)	Biological Function	Latent Viral Genes Expressed	Non-Coding RNAs
Latency III	Proliferation program	Naive B cells	Clonal expansion of infected naive B cells	EBNA1 EBNA2 EBNA3s (A,B,C) EBNA-LP LMP1 LMP2	EBERs BHRF1 miRNAs BART miRNAs
Latency II	Default program	B cells at the germinal-center differentiation stage	Escape from the primary immune response	EBNA1 LMP1 LMP2	EBERs BART miRNAs
Latency 0	Quiescence program	Resting memory B cells	Cell survival	/	EBERs BART miRNAs
Latency I	Maintenance program	Dividing memory B lymphocytes	Vertical transmission of the viral genome to daughter cells	EBNA1	EBERs BART miRNAs

BART miRNA: BamHI A rightward transcript microRNA; B cell: B lymphocyte; BHRF1 miRNA: BamHI fragment H rightward open reading frame 1 microRNA; EBER: Epstein–Barr encoded RNA; EBNA: Epstein–Barr Nuclear Antigen; EBNA-LP: Epstein–Barr Nuclear Antigen—Leader Protein; LMP: Latent Membrane Protein; RNA: ribonucleic acid.

**Table 3:** Summary of the key molecular functions associated with the EBV nuclear and membrane latency antigens. Adapted from Yu and Robertson (2023) [1].

Antigen Category	Antigen Name	Key Molecular Functions
Nuclear antigen	EBNA1	Maintenance and replication of the episomal genome (dsDNA) Inhibition of apoptosis Induction of genomic instability via production of reactive oxygen species (ROS) Transcriptional regulation of EBNAs (including EBNA1 itself) and of LMP1 via interaction with specific viral promoters Enables evasion from cytotoxic T-lymphocyte immune surveillance
Nuclear antigen	EBNA2	Essential transactivator for the activation of viral (EBNAs, LMPs) and cellular (e.g., MYC, CD23) genes Reprogramming of host gene regulatory programs Functionally substitutes for the intracellular domain of the Notch receptor
Nuclear antigen	EBNA-3A and EBNA-3C	Required for efficient B-lymphocyte transformation in vitro Inhibition of various cyclin-dependent kinase inhibitors (BIM, BLIMP-1, and p16/p15/p18 expression) Epigenetic control of gene transcription
Nuclear antigen	EBNA-3B	Functions as a tumor suppressor
Nuclear antigen	EBNA-LP	Assists EBNA2-mediated transcriptional activation Suppresses the innate cellular response triggered by viral DNA
Membrane antigen	LMP1	Principal EBV transforming antigen Mimics CD40 receptor signaling and activates the NF-κB, JAK/STAT, and other cellular signaling pathways Prevents p53-mediated apoptosis Promotes cell proliferation Activates signaling pathways involved in tumor invasion and metastasis Enables immune evasion via the PI3K-AKT-mTOR pathway
Membrane antigen	LMP2A	Mimics B-cell receptor (BCR) signaling to ensure B-lymphocyte survival and proliferation Impairs apoptosis and cell-cycle checkpoints by dysregulating key regulators Increases epithelial cell adhesion and motility via the PI3K-Akt pathway Dampens interferon receptor (IFNR) signaling to circumvent the innate immune response
Membrane antigen	LMP2B	Modulates the effects of LMP2A on the BCR

BCR: B-cell receptor; BIM: B-cell lymphoma 2-interacting mediator of cell death; BLIMP-1: B-lymphocyte-induced maturation protein 1; CD23: cluster of differentiation 23; dsDNA: double-stranded deoxyribonucleic acid; EBNA: Epstein–Barr Nuclear Antigen; IFNR: interferon receptor; JAK/STAT: Janus kinase/signal transducer and activator of transcription; LMP: latent membrane protein; MYC: myelocytomatosis oncogene; NF-κB: nuclear factor kappa-light-chain-enhancer of activated B cells; ROS: reactive oxygen species.

**Table 4:** Impact of EBV infection on the humoral B-cell and T-cell response [4].

Humoral Response	T-Cell Response
Patients with multiple sclerosis show markedly higher anti-EBNA1 antibody titers than EBV-positive individuals without MS.	No clear consensus in peripheral blood: reported frequencies of EBV-specific CD8+ T lymphocytes in MS patients vary across studies (decreased, unchanged, or increased) relative to EBV-positive healthy controls.
Anti-EBNA1 antibody titers predict the future risk of developing the disease and continue to rise after its onset.	At more advanced stages of MS, these CD8+ T lymphocytes show reduced functionality, suggesting a less effective antiviral response.
Anti-EBNA1 antibodies are detectable in the cerebrospinal fluid, reflecting an intrathecal humoral response.	The TCR repertoire is skewed toward the most immunogenic viral antigens (particularly EBNA1), with identical public clones detected in both blood and CSF across multiple MS patients, while such clones are absent or rare in EBV-positive healthy controls.
Antibodies against other viral antigens, such as VCA, EA-D, EBNA2, and EBNA3C, are likewise elevated in MS patients.	In the cerebrospinal fluid of MS patients, an enrichment of EBV-specific CD8+ T-cell clones is observed, exceeding that found in blood.
This humoral amplification is modulated by genetic factors: the HLA-DRB1*15:01 allele is associated with higher anti-EBNA1 titers, whereas the protective HLA-A*02:01 allele is associated with a more attenuated humoral response.	EBV-specific CD8+ T lymphocytes have been identified within active MS lesions.
	MS patients also show an increase in EBNA1-specific CD4+ T lymphocytes, with a pro-inflammatory Th1 profile.

CD4+: cluster of differentiation 4; CD8+: cluster of differentiation 8; EA-D: early antigen-diffuse; EBNA1: Epstein–Barr Nuclear Antigen 1; EBNA2: Epstein–Barr Nuclear Antigen 2; EBNA3C: Epstein–Barr Nuclear Antigen 3C; EBV: Epstein–Barr virus; HLA-A: human leukocyte antigen-A; HLA-DRB1: human leukocyte antigen-DR beta 1; MS: multiple sclerosis; TCR: T-cell receptor; Th1: T-helper cell type 1; VCA: viral capsid antigen.

**Table 5:** Summary of the main mechanisms by which EBV disrupts immune homeostasis [3,25,26].

Mechanism	Molecular Process	Diseases Illustrated
Molecular mimicry [25]	Structural homology between viral antigens and host proteins, leading to cross-reactive immune responses.	Rheumatoid arthritis Systemic lupus erythematosus
Bystander activation [26]	Release of cytokines (IL-17A) in an inflammatory context, activating non-specific lymphocytes.	Inflammatory bowel disease (Crohn’s disease and ulcerative colitis)
Epitope spreading [27]	Cell lysis releasing cryptic antigens that are recognized as neo-epitopes, amplifying the immune response.	IgA nephropathy
Epigenetic transactivation [3]	Binding of the latency protein EBNA2 to host risk loci, altering the expression of immune susceptibility genes.	Systemic lupus erythematosus Rheumatoid arthritis Crohn’s disease Type 1 diabetes Juvenile idiopathic arthritis Celiac disease

EBNA2: Epstein–Barr Nuclear Antigen 2; IgA: immunoglobulin A; IL-17A: interleukin 17A.

**Table 6:** Main lymphoproliferative lesions that may be associated with Epstein–Barr virus (EBV) infection, classified according to their virological status (consistently positive, sometimes positive) and immunosuppression context [5].

Lesion Type	EBV Status	Immunosuppression Context
Lymphomatoid granulomatosis	Distinct entity, consistently EBV-positive	No immunosuppression
EBV-positive diffuse large B-cell lymphoma	Distinct entity, consistently EBV-positive	No immunosuppression
Fibrin-associated large B-cell lymphoma	Distinct entity, consistently EBV-positive	Local immunosuppression
EBV-positive diffuse large B-cell lymphoma associated with chronic inflammation	Distinct entity, consistently EBV-positive	Local immunosuppression
Plasmablastic lymphoma	Generally considered EBV-positive, though rare EBV-negative cases occur	Variable immunosuppression
EBV-positive mucocutaneous ulcer	Distinct entity, consistently EBV-positive	Variable immunosuppression
EBV-positive polymorphic B-cell hyperplasia associated with immunosuppression	EBV-positive lesion (immunosuppression-related entity)	Systemic immunosuppression
EBV-positive polymorphic lymphoproliferative lesion associated with immunosuppression	EBV-positive lesion (immunosuppression-related entity)	Systemic immunosuppression
EBV-positive B-cell lymphoma associated with immunosuppression	EBV-positive lesion (immunosuppression-related entity)	Systemic immunosuppression
Reactive hyperplasia	Sometimes EBV-positive (non-distinct)	Variable immunosuppression
Classic Hodgkin lymphoma	Sometimes EBV-positive (non-distinct)	Variable immunosuppression
Marginal zone lymphoma	Sometimes EBV-positive (non-distinct)	Variable immunosuppression
Follicular lymphoma	Sometimes EBV-positive (non-distinct)	Variable immunosuppression
Plasmacytoma	Sometimes EBV-positive (non-distinct)	Variable immunosuppression

**Table 7:** Risk factors, epidemiological data, and histopathological classification of post-transplant lymphoproliferative disorders (PTLD), adapted from Puła et al. (2025) [9] and the 2022 WHO/ICC criteria.

Risk Factors and Epidemiological Data		
Risk Factor	Clinical Group	PTLD Statistics
Type of transplant	Solid organ transplantation Allogeneic hematopoietic stem cell transplantation	20% of post-transplant cancers 0.1–2.5% overall incidence
Nature of the transplanted tissue/organ	Multi-organ transplantation Intestinal transplantation Lung transplantation Heart transplantation Liver transplantation Kidney transplantation	12–33% * 20–30% 6–10% 3–5% 2–3% 1.5–2.5% * N.B.: except for combined kidney-pancreas transplantation (2–3%)
Donor–recipient HLA compatibility (allo-HSCT only)	HLA-mismatched unrelated donor HLA-matched unrelated donor Haploidentical donor HLA-matched related donor	11.2% 4.0% 2.8% 1.2%
Intensity of T-cell immunosuppression	Belatacept (anti-CTLA4 antibody) Efalizumab (anti-LFA1 antibody) Muromonab (anti-CD3 antibody) Alemtuzumab (anti-CD52 antibody) ATG (anti-thymocyte globulin) MMF (mycophenolate mofetil)	Increased PTLD risk Increased PTLD risk Increased PTLD risk Increased PTLD risk Increased PTLD risk No effect on PTLD risk
EBV serostatus	Donor+/Recipient– EBV serology mismatch	Significantly increased PTLD risk

allo-HSCT: allogeneic hematopoietic stem cell transplantation; ATG: anti-thymocyte globulin; CD3: cluster of differentiation 3; CD52: cluster of differentiation 52; CTLA4: cytotoxic T-lymphocyte-associated protein 4; EBV: Epstein–Barr virus; HLA: human leukocyte antigen; LFA1: lymphocyte function-associated antigen 1; MMF: mycophenolate mofetil; N.B.: nota bene; PTLD: post-transplant lymphoproliferative disease.* N.B.: except for combined kidney-pancreas transplantation (2–3%).

**Table 8:** Histopathological Classification and Clinical Features.

Histopathological Criterion	Subtypes	Relevant Clinical Information	Treatment
Non-destructive forms	Plasmacytic hyperplasia Infectious-mononucleosis-like syndrome with nodular hyperplasia	Early clinical forms occurring after solid organ transplantation (SOT) Occur mainly in children and EBV-naive adults before transplantation	Reduction of immunosuppression (RIS) alone as first-line treatment (low risk); close monitoring alone may suffice if regression occurs
Monomorphic form	B-cell lineage (majority of cases): diffuse large B-cell lymphoma, plasma cell neoplasms, others T/NK-cell lineage (rare cases)	Early clinical forms, often asymptomatic or paucisymptomatic Occur mainly in children and EBV-naive adults before transplantation	Risk-stratified sequential algorithm: RIS followed by weekly rituximab (induction); de-escalation if complete response, R-CHOP (4 cycles) if insufficient response
Polymorphic forms	/	Typical clinical presentation of lymphoproliferative malignancies Disease rarely confined to lymph nodes alone In late-onset forms, extranodal sites predominate: gastrointestinal tract (up to 30%) and central nervous system (5–20%) Possible complications: pneumonia, hepatitis, gastrointestinal motility disorders, cytopenia from marrow infiltration (sometimes the sole site), macrophage activation syndrome (HLH), or septic shock	Same sequential algorithm as the monomorphic form (RIS followed by rituximab, then R-CHOP according to response)
Classic Hodgkin lymphoma	/	Characterized by an infiltrate containing a small number of neoplastic cells Antigenic profile of tumor cells: obligatory expression of CD15 and CD30 Possible CD20 expression, but complete absence of CD3 and CD45 expression Tumor cells are surrounded by numerous inflammatory cells	Individualized management according to classic Hodgkin lymphoma protocols, combined with RIS

CD3: cluster of differentiation 3; CD15: cluster of differentiation 15; CD20: cluster of differentiation 20; CD30: cluster of differentiation 30; CD45: cluster of differentiation 45; EBV: Epstein–Barr virus; HLH: hemophagocytic lymphohistiocytosis (or macrophage activation syndrome); NK: natural killer; SOT: solid organ transplantation.

## Data Availability

No new data were created or analyzed in this study. Data sharing is not applicable to this article.

## Abbreviations

The following abbreviations are used in this manuscript:

allo-HSCT: allogeneic hematopoietic stem cell transplantation; CAEBV: chronic active Epstein–Barr virus disease; EBER: Epstein–Barr encoded RNA; EBNA: Epstein–Barr Nuclear Antigen; EBV: Epstein–Barr virus; HHV-4: human herpesvirus type 4; HLA: human leukocyte antigen; HLH: hemophagocytic lymphohistiocytosis; IBD: inflammatory bowel disease; IM: infectious mononucleosis; JIA: juvenile idiopathic arthritis; LMP: latent membrane protein; MS: multiple sclerosis; NK: natural killer (cell); PCR: polymerase chain reaction; PTLD: post-transplant lymphoproliferative disorder; RA: rheumatoid arthritis; SLE: systemic lupus erythematosus; SOT: solid organ transplantation; T1D: type 1 diabetes; TCR: T-cell receptor; UC: ulcerative colitis; VCA: viral capsid antigen; WHO/ICC: World Health Organization/International Consensus Classification; XLP: X-linked lymphoproliferative syndrome.

## References

[1] Yu H., Robertson E.S. (2023). Epstein–Barr Virus History and Pathogenesis. Viruses.

[2] Fugl A., Andersen C.L. (2019). Epstein-Barr virus and its association with disease—A review of relevance to general practice. BMC Fam. Pract..

[3] Morawiec N., Adamczyk B., Spyra A., Herba M., Boczek S., Korbel N., Polechoński P., Adamczyk-Sowa M. (2025). The Role of Epstein-Barr Virus in the Pathogenesis of Autoimmune Diseases. Medicina.

[4] Wahbeh F., Sabatino J.J. (2025). Epstein-Barr Virus in Multiple Sclerosis: Past, Present, and Future. Neurol. Neuroimmunol. Neuroinflamm..

[5] Marsh R.A. (2018). Epstein-Barr Virus and Hemophagocytic Lymphohistiocytosis. Front. Immunol..

[6] Yildiz H., Van Den Neste E., Defour J.P., Danse E., Yombi J. (2022). Adult haemophagocytic lymphohistiocytosis: Review. QJM.

[7] Chapman J. (2023). Immunodeficiency-associated Epstein-Barr virus-positive B-cell lymphoproliferative disorders: A review and new paradigm. Surg. Pathol..

[8] Gold J.E., Okyay R.A., Licht W.E., Hurley D.J. (2021). Investigation of long COVID prevalence and its relationship to Epstein-Barr virus reactivation. Pathogens.

[9] Puła B., Drozd-Sokołowska J., Czyż A., Giebel S., Lech-Marańda E., Gil L., Wróbel T., Styczyński J., Zaucha J.M. (2025). Post-transplant lymphoproliferative diseases: Polish Lymphoma Research Group 2025 recommendations for diagnosis and treatment. Acta Haematol. Pol..

[10] Kimura H., Cohen J.I. (2026). Chronic active Epstein-Barr virus disease: Molecular pathogenesis, evolving concepts, and emerging therapies. Blood.

[11] International Agency for Research on Cancer (IARC), World Health Organization Nasopharynx: Global Cancer Observatory (GLOBOCAN) 2022 Fact Sheet. https://gco.iarc.who.int/media/globocan/factsheets/cancers/4-nasopharynx-fact-sheet.pdf.

[12] Hirabayashi M., Georges D., Clifford G.M., de Martel C. (2023). Estimating the Global Burden of Epstein-Barr Virus-Associated Gastric Cancer: A Systematic Review and Meta-Analysis. Clin. Gastroenterol. Hepatol..

[13] Lupton S., Levine A.J. (1985). Mapping genetic elements of Epstein-Barr virus that facilitate extrachromosomal persistence of Epstein-Barr virus-derived plasmids in human cells. Mol. Cell. Biol..

[14] Frappier L. (2012). The Epstein-Barr virus EBNA1 protein. Scientifica.

[15] Jiang L., Xie C., Lang J., Lung H.L., Lo K.W., Law G.-L., Mak N.-K., Wong K.-L. (2018). EBNA1-targeted inhibitors: Novel approaches for the treatment of Epstein-Barr virus-associated cancers. Theranostics.

[16] Messick T.E., Smith G.R., Soldan S.S., McDonnell M.E., Deakyne J.S., Malecka K.A., Tolvinski L., van den Heuvel A.P.J., Gu B.-W., Cassel J.A. (2019). Structure-based design of small molecule inhibitors of Epstein-Barr virus EBNA1 DNA binding. Sci. Transl. Med..

[17] Colevas A.D., Talebi Z., Winters E., Even C., Lee V.H.-F., Gillison M.L., Khan S.A., Lu R., Pinsky B.A., Soldan S.S. (2025). First-in-human clinical trial of a small molecule EBNA1 inhibitor, VK-2019, in patients with Epstein-Barr positive nasopharyngeal cancer, with pharmacokinetic and pharmacodynamic studies. Clin. Cancer Res..

[18] García-Peris M., Jiménez Candel M.I., Mañes Jiménez Y., Pariente Martí M., González Granda D., Calvo Rigual F. (2019). Epstein-Barr virus primary infection in healthy children. An. Pediatr..

[19] Rostgaard K., Balfour H.H., Jarrett R., Erikstrup C., Pedersen O., Ullum H., Nielsen L.P., Voldstedlund M., Hjalgrim H. (2019). Primary Epstein-Barr virus infection with and without infectious mononucleosis. PLoS ONE.

[20] Murray T.J. (2009). The history of multiple sclerosis: The changing frame of the disease over the centuries. J. Neurol. Sci..

[21] Bjornevik K., Cortese M., Healy B.C., Kuhle J., Mina M.J., Leng Y., Elledge S.J., Niebuhr D.W., Scher A.I., Munger K.L. (2022). Longitudinal analysis reveals high prevalence of Epstein-Barr virus associated with multiple sclerosis. Science.

[22] Lanz T.V., Brewer R.C., Ho P.P., Moon J.-S., Jude K.M., Fernandez D., Fernandes R.A., Gomez A.M., Nadj G.-S., Bartley C.M. (2022). Clonally expanded B cells in multiple sclerosis bind EBV EBNA1 and GlialCAM. Nature.

[23] Rotstein D.L., Maxwell C., Tremlett H., Vyas M.V., Everett K., Marrie R.A. (2026). Multiple Sclerosis Onset Preceding Epstein-Barr Virus Infection. JAMA Neurol..

[24] Vietzen H., Kühner L.M., Berger S.M., Reinecke R., Rostásy K., Saucke H., Kauth F., Koukou G., Sommer S., Wendel E.-M. (2026). Epstein-Barr Virus Antibodies to Differentiate Multiple Sclerosis From Other Neuroinflammatory Diseases. JAMA Neurol..

[25] Munir M. (2025). The role of Epstein-Barr virus molecular mimicry in various autoimmune diseases. Scand. J. Immunol..

[26] Nour Eddine H.F., Kassem A.M., Salhab Z., Sherri N., Moghabghab K., Mohsen Z., Naim G., Mahmoud S., Jurjus A., Hashash J.G. (2025). Toll-like receptor 9 mediates Epstein-Barr virus-aggravated inflammation in a mouse model of inflammatory bowel disease. Biomedicines.

[27] Mestecky J., Julian B.A., Raska M. (2023). IgA nephropathy: Pleiotropic impact of Epstein-Barr virus infection on immunopathogenesis and racial incidence of the disease. Front. Immunol..

[28] Younis S., Moutusy S.I., Rasouli S., Jahanbani S., Pandit M., Wu X., Acharya S., Sharpe O., Wijeratne T.U., Harris M.L. (2025). Epstein-Barr virus reprograms autoreactive B cells as antigen-presenting cells in systemic lupus erythematosus. Sci. Transl. Med..

[29] Robinson W.H., Younis S., Love Z.Z., Steinman L., Lanz T.V. (2024). Epstein–Barr virus as a potentiator of autoimmune diseases. Nat. Rev. Rheumatol..

[30] Wu Y., Sun X., Kang K., Yang Y., Li H., Zhao A., Niu T. (2024). Hemophagocytic lymphohistiocytosis: Current treatment advances, emerging targeted therapy and underlying mechanisms. J. Hematol. Oncol..

[31] Kong Q., Li M., Wang J., Wu L., Zhou D., Yang M., Xu X., Tan Z., Wu X., Wang Z. (2024). Prognostic scoring system for pediatric Epstein–Barr virus-associated hemophagocytic lymphohistiocytosis based on baseline characteristics: A multicenter retrospective study. Pediatr. Blood Cancer.

[32] Shamriz O., Kumar D., Shim J., Briones M., Quarmyne M., Chonat S., Lucas L., Edington H., White M.H., Mahajan A. (2021). T Cell-Epstein-Barr Virus–Associated Hemophagocytic Lymphohistiocytosis (HLH) Occurs in Non-Asians and Is Associated with a T Cell Activation State that Is Comparable to Primary HLH. J. Clin. Immunol..

[33] El-Mallawany N.K., Curry C.V., Allen C.E. (2022). Haemophagocytic lymphohistiocytosis and Epstein-Barr virus: A complex relationship with diverse origins, expression and outcomes. Br. J. Haematol..

[34] Bergsten E., Horne A., Aricò M., Astigarraga I., Egeler R.M., Filipovich A.H., Ishii E., Janka G., Ladisch S., Lehmberg K. (2017). Confirmed efficacy of etoposide and dexamethasone in HLH treatment: Long-term results of the cooperative HLH-2004 study. Blood.

[35] Wang W., Zhao Y., Ge J., Fang Z., Zhou C., Wang D., Zhang Q., Li Z., Wang T., Zhang R. (2025). Efficacy and safety of ruxolitinib-based regimen in the treatment of paediatric Epstein-Barr virus-associated haemophagocytic lymphohistiocytosis. Br. J. Haematol..

[36] Luniewski A., Chaudhary S., Goldfarb A., Obiorah I.E. (2026). EBV-Driven NK/T-Cell Lymphoproliferative Disorders: Clinical Diversity and Molecular Insights. Lymphatics.

[37] Orlando E.H., Gould P., Cuzzo B., Ford M., Chen Y., Sanjurjo A., Jain S., May B., Tsapepas D., Leeman-Neill R.J. (2025). Posttransplant rituximab exposure and risk of PTLD in solid organ transplant recipients with EBV DNAemia. Blood Neoplasia.

[38] European Medicines Agency (EMA) (2022). Ebvallo, INN-Tabelecleucel: EPAR—Product Information. https://www.ema.europa.eu/en/documents/product-information/ebvallo-epar-product-information_en.pdf.

[39] Uemura Y., Yamamoto M., Ishimura M., Kanegane H., Sawada A., Hirakawa A., Imadome K.-I., Yoshimori M., Nagata M., Yamamoto K. (2025). JAK1/2 inhibitor ruxolitinib for the treatment of systemic chronic active Epstein-Barr virus disease: A phase 2 study. Blood Neoplasia.

[40] Harada N., Sonoda M., Park S., Ohga S., Eguchi K., Adachi S., Kinoshita K., Yada Y., Shiraishi A., Ohshima K. (2026). Long-term outcomes of chronic active Epstein–Barr virus infection in childhood after dominantly infected lymphocyte subpopulation management. J. Infect. Dis..

